# Medical Utilization of Emergency Departments among Patients with Prostate Cancer: A Nationwide Population-Based Study in Taiwan

**DOI:** 10.3390/ijerph182413233

**Published:** 2021-12-15

**Authors:** Jui-Ming Liu, Ren-Jun Hsu, Yu-Tang Chen, Yueh-Ping Liu

**Affiliations:** 1 Department of Urology, Taoyuan General Hospital, Ministry of Health and Welfare, Taoyuan 330, Taiwan; mento1218@gmail.com; 2 Department of Obstetrics and Gynecology, Tri-Service General Hospital, National Defense Medical Center, Taipei 114, Taiwan; 3 Cancer Center, Hualien Tzu Chi Hospital, Buddhist Tzu Chi Medical Foundation, Hualien 970, Taiwan; hsurnai@gmail.com (R.-J.H.); ytc1138@gmail.com (Y.-T.C.); 4 College of Medicine, Tzu Chi University, Hualien 970, Taiwan; 5 Department of Emergency Medicine, National Taiwan University Hospital, Taipei 100, Taiwan; 6 Department of Medical Affairs, Ministry of Health and Welfare, Taipei 115, Taiwan

**Keywords:** prostate cancer, emergency department, National Health Insurance Research Database

## Abstract

(1) Background: In Taiwan, prostate cancer is a major malignancy with an increasing incidence among men. This study explores the medical utilization of emergency departments among patients with prostate cancer in Taiwan. (2) Methods: This nationwide, population-based study was conducted via a cross-sectional method based on the Registry for Catastrophic Illness Patient Database from Taiwan’s National Health Insurance Research Database. Patients with newly diagnosed prostate cancer between 1997 and 2013 were enrolled in the study and divided into four treatment-related groups. The rate of emergency department presentation, disease categorization of emergency department visits, emergency department-related medical expenditures, and temporal trends were investigated. (3) Results: A total of 18,728 patients with prostate cancer were identified between 1997 and 2013, for whom 13,098 emergency department visits were recorded. The number of emergency department visits increased during the study period. The incidence rate for the medical utilization of emergency department visits was 822 per 1000 people during the study period. The incidence rates for patients with prostate cancer in the radical prostatectomy, radiotherapy, androgen deprivation therapy, and chemotherapy groups were 549, 1611, 1101, and 372, respectively. The average medical expenditure per emergency department visit was TWD 3779.8 ± 5116.2, and the expenditure was recorded for the chemotherapy group at TWD 4690.8 ± 7043.3. The most common disease diagnoses among patients with prostate cancer who presented to the emergency department were injury/poisoning (16.79%), genitourinary disorders (10.66%), and digestive disorders (10.48%). (4) Conclusions: This nationwide population-based study examined the emergency department visits of patients with prostate cancer in Taiwan, providing useful information for improving the quality of medical care.

## 1. Introduction

Prostate cancer (PCa) represents the most common cancer affecting men in the US and is the fifth most common cancer among men in Taiwan [1,2]. According to The Global Cancer Observatory: CANCER TODAY (GLOBOCAN), more than 1.1 million patients were newly diagnosed with PCa, and 330,000 deaths occurred among patients with PCa in 2012 [3]. In Taiwan, PCa incidence has increased yearly up to 31.7 per 100,000 persons, with 6644 new PCa diagnoses and 1377 deaths reported in 2017 [4]. In a recent report, the annual expenditures associated with cancer treatments were estimated to reach USD 173 billion in the year 2020 [5]. These reports confirm the high incidence and high expenditures associated with PCa care worldwide.

The standard PCa treatment depends on the clinical tumor stage and can include surgery in the form of radical prostatectomy (RP); radiotherapy (RT); androgen deprivation therapy (ADT); or chemotherapy (CT) [6]. The incidence of advanced PCa has increased with the aging population, and the median age at the time of PCa diagnosis was 72 years in the Taiwanese population [4]. Existing PCa treatment options are associated with various adverse effects, including intolerable pain, hematuria, or urine retention, which can drive patients to emergency departments (EDs) seeking medical help. In addition, some castration-resistant patients with PCa who undergo CT may visit the ED seeking palliative care, such as pain relief. Patients may also require services from other providers or the ED for unexpected health concerns unrelated to PCa.

In Taiwan, the National Health Insurance (NHI) system is available to all residents through a low insurance tax, making medical utilization easily accessible. Based on the records of the NHI system, each patient attended 13.4 physician consultations, including 3.4 specialty consultations, each year [7]. PCa is a major public health issue, and providing better care to patients with PCa to meet their medical needs is an important goal. Patients with PCa are able to easily access medical facilities or the ED if they suffer from intolerable medical problems.

Recognizing the epidemiological characteristics associated with the medical utilization of EDs among patients with PCa, including the associated medical problems and medical expenditure, may provide us with important information for how to improve PCa care. This retrospective, nationwide, population-based study used data obtained from Taiwan’s National Health Insurance Research Database (NHIRD) to assess the general characteristics of ED visits among patients with PCa in Taiwan, including the underlying medical reasons detailed divided into different categories according to the ICD-9-CM codes including infectious and parasitic diseases; neoplasms; endocrine disorders; nutritional and metabolic diseases and immunity disorders; blood; mental disorders; the nervous system and sensory organs; the circulatory system; the respiratory system; the digestive system; the genitourinary system; the skin and subcutaneous tissue; the musculoskeletal system and connective tissue; symptoms, signs, and ill-defined conditions; and injury.

## 2. Materials and Methods

### 2.1. Database

This retrospective study used the Registry for Catastrophic Illness Patient Database (RCIPD), which is a sub-database of the NHIRD of Taiwan. The NHIRD is an administrative database derived from the National Health Insurance (NHI) program, the national medical insurance system of Taiwan, which has enrolled greater than 99% of all Taiwan residents. The RCIPD includes the healthcare data for patients diagnosed with catastrophic illnesses, such as those with malignancy, trauma, or severe immune and mental illnesses. Patients can obtain a certification of catastrophic illness after being reviewed by the experts of the NHI program. Therefore, we can access the medical information of PCa patients in the RCIPD [8,9]. The diagnostic codes associated with patients in the RCIPD and NHIRD are based on the International Classification of Diseases, 9th revision, Clinical Modification (ICD-9-CM). This study was approved by the Institutional Review Board of the Tri-Service General Hospital (approval number: TSGHIRB NO B-104-21).

### 2.2. Study Population

**Figure 1:**
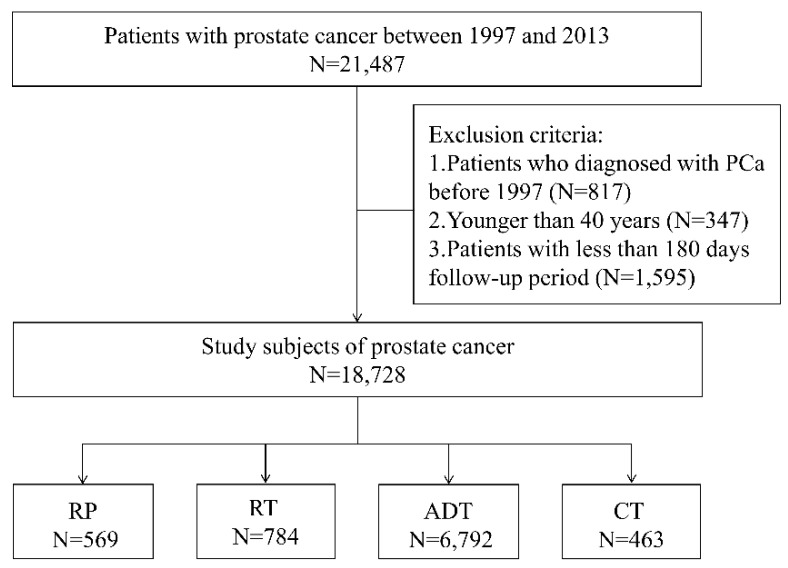
Study flowchart. RP, radical prostatectomy; RT, radiation therapy; ADT, androgen deprivation therapy; CT, chemotherapy.

All patients with PCa older than 40 years in the RCIPD database were enrolled in this study. The study period was between 1997 and 2013, and all patients who received a new diagnosis of PCa during the study period were included. The accuracy of the PCa diagnosis was high for all patients, and enrollment in the RCIPD was certified by NHI experts. The exclusion criteria for this study were patients diagnosed with PCa before 1997, patients younger than 40 years, and patients with less than 180 days’ follow-up. The study subjects were further divided into four groups according to PCa treatment. Those who only received RP were defined as the RP group; those who only received RT were defined as the RT group; those who currently received ADT were defined as the ADT group; and those who currently received CT were defined as the CT group listed in Figure 1.

Information regarding ED visits by patients with PCa is also available from the RCIPD. The medical diagnoses associated with ED visits were made by ED physicians and were further divided into several categories according to the ICD-9-CM codes, including 001–139 infectious and parasitic diseases; 140–239 neoplasms; 240–279 endocrine disorders; nutritional and metabolic diseases and immunity disorders; 280–289 diseases of the blood and blood-forming organs; 290–319 mental disorders; 320–389 diseases of the nervous system and sensory organs; 390–459 diseases of the circulatory system; 460–519 diseases of the respiratory system; 520–579 diseases of the digestive system; 580–629 diseases of the genitourinary system; 680–709 diseases of the skin and subcutaneous tissue; 710–739 diseases of the musculoskeletal system and connective tissue; 780–799 symptoms, signs, and ill-defined conditions; 800–999 injury; and poisoning. [10] The total cost of ED visit expenditures was calculated in Taiwan New Dollars (TWD); the exchange rate is TWD 28 for USD 1. We collected and analyzed information on ED visits each month, including disease categories, expenditures, and epidemiological characteristics.

### 2.3. Statistical Analysis

We used the software programs SPSS version 22.0 for Windows (IBM, Armonk, NY, USA) and SAS version 9.2 (SAS Institute, Cary, NC, USA) for descriptive data management. Descriptive statistics are represented as the number of study subjects, percentage, and mean with standard deviation (SD). The incidence rates (IR, per 1000 peope/year), were calculated and analyzed using the software program STATA version 11.2 (StataCorp, College Station, TX, USA). *p* < 0.05 was defined as significant.

## 3. Results

We identified 21,487 patients with PCa from the RCIPD between 1997 and 2013, including 18,728 patients who were selected for study inclusion after reviewing the study criteria. The ED visits by the selected patients were determined by reviewing RCIPD data. The overall ED utilization among patients with PCa is displayed in Table 1. A total of 13,098 patients with PCa visited the ED during the study period, including a total of 74,075 ED visits during the treatment follow-up period, and the number of ED visits increased steadily from 1997 to 2013, as displayed in Figure S1.

The mean expenditure per ED visit was TWD 3779.82. The ED utilization according to the PCa treatment group is displayed in Table 2. The numbers of patients in each group were 569; 784; 6792; and 463 for the RP, RT, ADT, and CT groups, respectively. The percentages of patients who visited the ED during the study period according to the treatment group were 55.71%, 74.49%, 76.40%, and 90.71%, for the RP, RT, ADT, and CT groups, respectively. The CT group included the highest percentage of patients who utilized the ED during the study period. The IR of ED visits per 1000 peope/year, from high to low, were 1611 in the RT group; 1372 in the CT group; 1101 in the ADT group; and 549 in the RP group. The mean medical expenditures per ED visit, from highest to lowest, were TWD 4690 for the CT group; TWD 3906 for the ADT group; TWD 3636 for the RP group; and TWD 3214 for the RT group.

**Figure 2:**
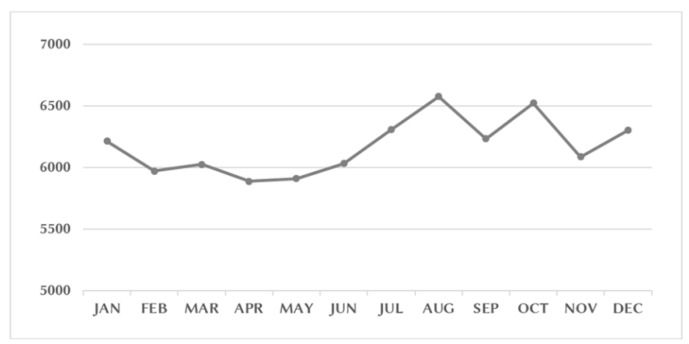
The monthly ED visits of prostate cancer patients from January 1997 to December 2013.

The distributions of diagnostic categories associated with ED visits among patients with PCa are listed in Table 3. With the exception of ill-defined symptoms or signs, the most common reasons that patients with PCa visited the ED were injury/poisoning (16.79%), genitourinary disorders (10.66%), and digestive disorders (10.48%). The monthly ED visits of PCa patients are shown in Figure 2. Increased numbers of ED visits were noted in August and October. The distribution of monthly ED visits according to the different PCa treatment groups is displayed in Figure S2. We observed increased numbers of ED visits in August among the RP, RT, and CT groups. An increase in October was observed for the ADT group.

## 4. Discussion

To the best of our knowledge, this is the first large, nationwide, population-based study to evaluate the medical utilization of the ED among patients with PCa in Taiwan. A total of 18,728 patients with PCa were enrolled in this study from the RCIPD between 1997 and 2013. Across all study subjects, 74,057 ED visits were recorded, with an increasing trend in the number of visits during the study period. The overall IR for ED visits was 822 per 1000 people/year, with higher IRs noted for the RT and CT groups. The mean medical expenditure was TWD 3779.8, with the highest expenditure reported for the CT group. The three most common medical illnesses diagnosed among patients with PCa during ED visits were injury/poisoning (16.79%), genitourinary disorders (10.66%), and digestive disorders (10.48%).

A meta-analysis reviewed ED use among cancer patients, which demonstrated that the percentage of cancer patients with ED visits ranged from 1 to 83% [11], including 3–5% of patients with PCa [12,13]. The 30-day visit rates were also noted as 2–12% after surgery and 5% after chemotherapy. In the current study, 13,098 patients with PCa visited the ED seeking medical help, representing 69.9% of all patients enrolled in the study and accounting for a total of 74,057 ED visits during the study period. We observed a higher rate of ED visits for patients with PCa in Taiwan, which might be due to the low health insurance tax associated with the NHI system and the easy access to medical facilities in Taiwan. In addition, PCa is among the major diseases that can be certified as a catastrophic illness, which entitles the patient to a medical payment waiver [14]. Yang et al. conducted a large population study and reported that older individuals in Taiwan diagnosed with catastrophic illnesses averaged 61.62 ED visits per 1000 people/month [10]. The ED visit IR of 822 per 1000 person-years from this study is compatible with the rate reported by the previous study. We also observed that the average medical expenditure associated with ED visits was TWD 3779.8, although the highest expenditure was observed for the CT group, at TWD 4690.8, which is similar to the results reported by Yang et al. of TWD 4367.2 among patients diagnosed with catastrophic illness [10]. The medical expenditures reported in Taiwan are much lower than those reported for Western countries [15,16] due to the low health insurance tax associated with the NHI system and the waiver for medical payments among those with certified catastrophic illnesses.

Another observational study of 3347 colon cancer patients between 2000 and 2012 in Taiwan demonstrated that the number of visits increased steadily from 2000 to 2012 [17]. This increasing trend in ED visits was also observed in the current study. Both colon cancer and PCa are major cancers with an increasing incidence in Taiwanese men [4]. A yearly increase in ED use by patients with PCa patients is a reasonable outcome. The guidelines for PCa management include multimodal treatment, which can comprise surgery, radiation therapy, hormone therapy, and chemotherapy. [6] Patients may suffer from various types and grades of adverse effects after PCa treatment. A nationwide study in the US enrolled 696 million ED visits from the Nationwide Emergency Department Sample, which revealed that PCa was the second most common cancer associated with an ED visit [18]. The most common primary reasons for cancer-related ED visits were pneumonia, nonspecific chest pain, and urinary tract infections. Among patients with PCa, genitourinary symptoms and urinary tract infections were the most common reasons for cancer-related ED visits [18]. In the current study, the three most common medical illnesses diagnosed at ED visits were injury/poisoning (16.79%), genitourinary disorders (10.66%), and digestive disorders (10.48%). Genitourinary disorders were also a major cause of ED visits among patients with PCa. Emerging data on the presentation of cancer populations to the ED suggest that different cancer types require different forms of medical assistance at ED visits [19,20,21,22]. In this study, a higher rate of ER visits in August, October, and January was observed. This phenomenon was also observed in a previous population study of Taiwan. Yang et al. enrolled 46,057 males and 50,854 females with catastrophic illness who showed an increase in ED visits in October, January, and February [10]. Further study remains necessary to better describe the ED visits associated with cancer and identify common cancer-related complications to optimize the use of clinical and ED care.

A strength of the present study was the use of a nationwide epidemiological survey to analyze ED visits among the Taiwanese population of patients with PCa. This study provides the first nationwide description of patients with PCa seeking medical care at the ED due to the manifestation of cancer-related symptoms. However, this study was also associated with several limitations. First, detailed histories and laboratory tests were not available through the NHIRD. Therefore, further risk assessments and analyses of risk factors could not be conducted. Second, patients with minor injuries who seek medical aid at outpatient clinics rather than the ED were not included in this study. Third, the NHIRD is organized according to the ICD-9-CM codes used. Physicians in EDs may omit codes or use the wrong codes, complicating the ability to perform a detailed investigation into the reasons for ED visits. Several possible confounders were not controlled for in the present study, including socioeconomic influences and family support, which could not be extracted from the NHIRD. Fourth, the Gleason score, prostate-specific antigen levels, and PCa stage are not available in the NHIRD, which made investigating associations between tumor stage and ED visits impossible to perform. Further studies are warranted to validate and elucidate the epidemiology of ED visits by patients with PCa.

## 5. Conclusions

This nationwide population-based study provided epidemiological evidence regarding the reasons patients with PCa visit the ED in Taiwan. An increasing trend in ED visits was observed among patients with PCa. The three most common reasons for ED visits were injury/poisoning, genitourinary disorders, and digestive disorders. Further study remains necessary to better describe why patients with PCa visit the ED in order to improve patient care.

## Figures and Tables

**Table 1:** Utilization of emergency department among patient with prostate cancer.

	Patients with PCa	
Total N (%)	18,728	(100)
ED-visited subjects (%)	13,098	(69.94)
Total number of ED visits	74,075	
Mean of ED visits	5.66	
IR of ED visits	822	
Mean (SD) of total expenditure per visit by TWD	3779.8 (5116.22)	

ED, Emergency department; IR, incidence rate per 1000 people/year; TWD, Taiwan New Dollars; SD, Standard deviation.

**Table 2:** Utilization of emergency department according to different treatment of prostate cancer.

	RP Group		RT Group		ADT Group		CT Group		*p* Value
Total N (%)	569	(100)	784	(100)	6792	(100)	463	(100)	
ED-visited subjects (%)	317	(55.71)	584	(74.49)	5189	(76.40)	420	(90.71)	
Total number of ED visits	1272		5021		29,223		2643		
Mean of ED visits	4.01		8.60		5.63		6.29		
IR of ED visits	549		1611		1101		1372		
Mean (SD) of total expenditure per visit by TWD	3637.0 (4935.97)		3214.4 (5313.34)		3906.9 (5134.38)		4690.9 (7043.31)		<0.001 **

** *p* < 0.001 ADT, androgen deprivation therapy; CT, chemotherapy; ED, Emergency department; IR, incidence rate per 1000 people/year; RP, radical prostatectomy; RT, radiation therapy; SD, Standard deviation; TWD, Taiwan New Dollars.

**Table 3:** Distribution of diagnostic categories of ED visits among patients with prostate cancer.

ICD-9 Codes	Diagnostic Category	All Prostate Cancer		RP (N = 569)		RT (N = 784)		ADT (N = 6792)		CT (N = 463)	
		No.	(%)	No.	(%)	No.	(%)	No.	(%)	No.	(%)
001–139	Infectious and parasitic diseases	295	0.86	8	1.14	12	0.73	108	0.77	9	0.70
140–239	Neoplasms	1712	4.96	34	4.86	193	11.75	945	6.75	192	14.90
240–279	Endocrine, nutritional and metabolic diseases, and immunity disorders	649	1.88	6	0.86	37	2.25	293	2.09	37	2.87
280–289	Diseases of the blood and blood-forming organs	310	0.90	2	0.29	27	1.64	162	1.16	45	3.49
290–319	Mental disorders	254	0.74	5	0.72	11	0.67	105	0.75	7	0.54
320–389	Diseases of the nervous system and sense organs	791	2.29	13	1.86	31	1.89	267	1.91	31	2.40
390–459	Diseases of the circulatory system	2859	8.29	50	7.15	114	6.94	1138	8.13	70	5.43
460–519	Diseases of the respiratory system	2989	8.66	42	6.01	139	8.46	1195	8.53	102	7.91
520–579	Diseases of the digestive system	3614	10.48	76	10.87	163	9.92	1449	10.35	122	9.46
580–629	Diseases of the genitourinary system	3677	10.66	68	9.73	168	10.23	1565	11.17	139	10.78
680–709	Diseases of the skin and subcutaneous tissue	925	2.68	23	3.29	44	2.68	335	2.39	27	2.09
710–739	Diseases of the musculoskeletal system and connective tissue	1391	4.03	31	4.43	63	3.83	563	4.02	52	4.03
780–799	Symptoms, signs, and ill-defined conditions	8785	25.46	179	25.61	390	23.74	3571	25.50	301	23.35
800–999	Injury and poisoning	5792	16.79	155	22.17	229	13.94	2150	15.35	142	11.02

ADT, androgen deprivation therapy; CT, chemotherapy; RP, radical prostatectomy; RT, radiation therapy.

## Supplementary Materials

The following are available online at https://www.mdpi.com/article/10.3390/ijerph182413233/s1, Figure S1: The number of ED visits of prostate cancer by year from 1997 to 2013, Figure S2: The distribution of monthly ED visits according to the different treatment groups of prostate cancer.
