# Supplementary material for: Medical Utilization of Emergency Departments among Patients with Prostate Cancer: A Nationwide Population-Based Study in Taiwan
Source: Int J Environ Res Public Health. 2021 Dec 15;18(24):13233. doi: 10.3390/ijerph182413233 (PMC8701110; doi:10.3390/ijerph182413233)
Supplement: Supplementary file 1 [file ijerph-18-13233-s001.zip › ijerph-1438245-supplementary.pdf]

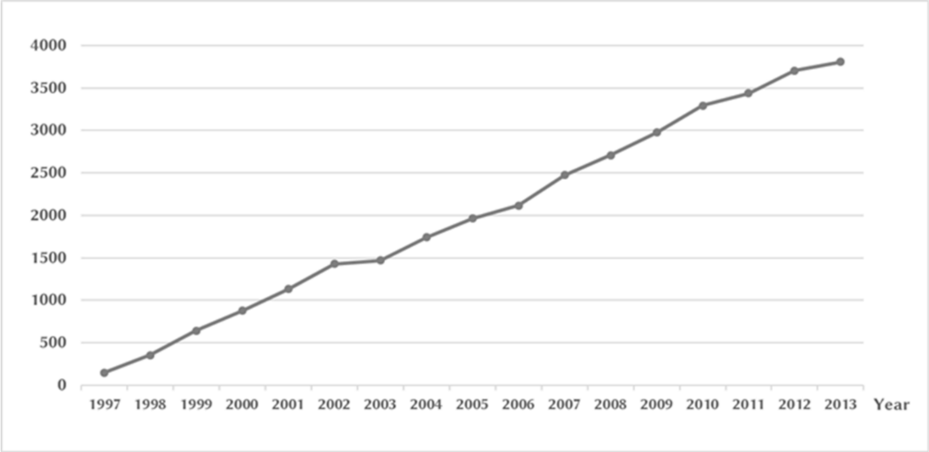

Figure S1. The yearly ED visits of prostate cancer patients from January 1997 to December 2013.

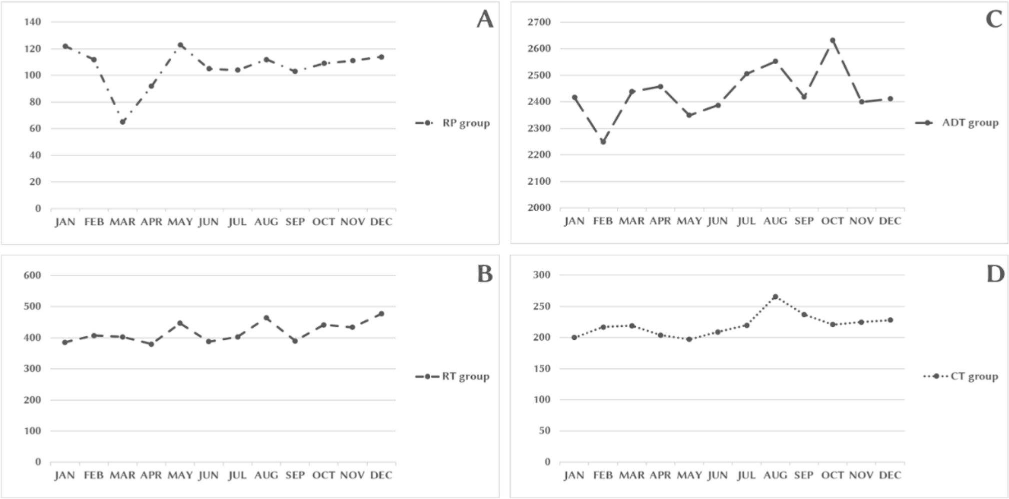

Figure S2. The distribution of monthly ED visits according to the different PCa treatment. (A)radical prostatectomy,RP (B)radiotherapy,RT (C)androgen deprivation therapy, ADT (D)chemotherapy, CT.
